# Dissecting the molecular mechanisms underlying the antidepressant activities of herbal medicines through the comprehensive review of the recent literatures

**DOI:** 10.3389/fpsyt.2022.1054726

**Published:** 2022-12-22

**Authors:** Yilu Sun, Jia Zhao, Jianhui Rong

**Affiliations:** ^1^Department of Chinese Medicine, The University of Hong Kong-Shenzhen Hospital, Shenzhen, China; ^2^School of Chinese Medicine, The University of Hong Kong, Pokfulam, Hong Kong SAR, China

**Keywords:** depression, molecular mechanisms, herbal medicines, active constituents, antidepressant

## Abstract

Depression is clinically defined as a mood disorder with persistent feeling of sadness, despair, fatigue, and loss of interest. The pathophysiology of depression is tightly regulated by the biosynthesis, transport and signaling of neurotransmitters [e.g., serotonin, norepinephrine, dopamine, or γ-aminobutyric acid (GABA)] in the central nervous system. The existing antidepressant drugs mainly target the dysfunctions of various neurotransmitters, while the efficacy of antidepressant therapeutics is undermined by different adverse side-effects. The present review aimed to dissect the molecular mechanisms underlying the antidepressant activities of herbal medicines toward the development of effective and safe antidepressant drugs. Our strategy involved comprehensive review and network pharmacology analysis for the active compounds and associated target proteins. As results, 45 different antidepressant herbal medicines were identified from various *in vivo* and *in vitro* studies. The antidepressant mechanisms might involve multiple signaling pathways that regulate neurotransmitters, neurogenesis, anti-inflammation, antioxidation, endocrine, and microbiota. Importantly, herbal medicines could modulate broader spectrum of the cellular pathways and processes to attenuate depression and avoid the side-effects of synthetic antidepressant drugs. The present review not only recognized the antidepressant potential of herbal medicines but also provided molecular insights for the development of novel antidepressant drugs.

## 1 Introduction

Depression is a common mental disease that seriously affects 5% of adults worldwide, especially postpartum women (1, 2). Diagnostic and statistical manual of mental disorders (DSM-5) divides depression disorder into eight categories: disruptive mood dysregulation disorder, major depressive disorder (including major depressive episode), persistent depressive disorder (dysthymia), premenstrual dysphoric disorder, substance/medication-induced depressive disorder, depressive disorder due to another medical condition, other specified depressive disorder, and unspecified depressive disorder (3). Patients with depression usually suffer from symptoms such as depressed mood, anxiety, loss of interest, lack of energy, pessimism, disappointment, self-denial and even suicidal thoughts, while 41% of depressed mothers may intend to harm their babies (4). Depression not only represents an ongoing medical challenge but also has emerged as a financial burden for global healthcare systems, for example, annual cost of nearly $210.5 billion in the United States (5). The existing treatments mainly alleviate depressive symptoms so that the remission rate is less than 60% (6). Most of antidepressant drugs cause different apparent adverse side-effects, resulting in the average withdrawal incidence rate of 56% (7, 8). Depression is well-known to be a multifactorial mental disease and exhibit various symptoms including sadness, anxiety, anger and irritability. Synthetic antidepressants are challenged by efficacy and severe side effects. Current first-line antidepressants like SSRIs and SNRIs are designed to specifically target the actions of serotonin and noradrenaline so that SSRIs and SNRIs may not be effective against depression as the result of multiple other causes (9). Thus, single-target therapies may fail in the treatment of multifactorial disease.

Nevertheless, 2.39–40% of patients in different countries and regions alternatively used herbal medicines (10–13). Encouragingly, traditional Chinese medicine (TCM) has achieved the effective use of herbal medicines to treat depression over thousands of years (14). Therefore, herbal medicines may serve as a rich source for the development of novel antidepressant therapies. These results stimulated us to examine the current understanding on the pathology of depression, the pharmacology of the existing antidepressant drugs and the antidepressant activity of herbal medicines toward the development of novel effective and safe antidepressant drugs.

## 2 Current understanding of depression

**FIGURE 1:**
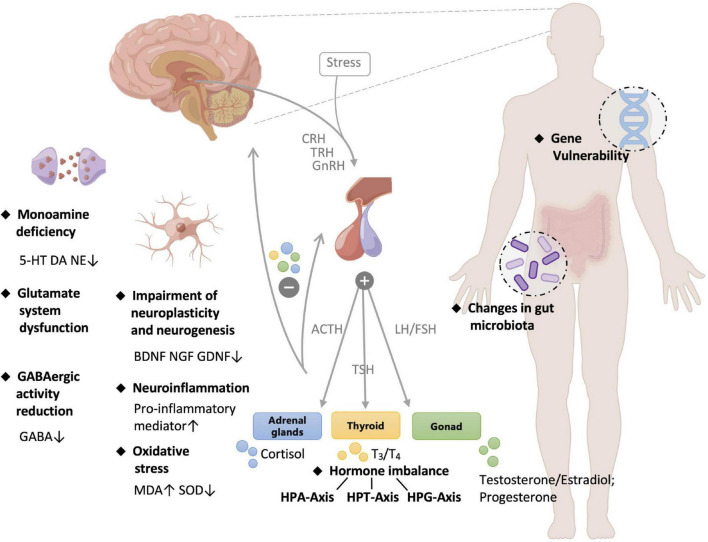
Pathology of depression. 5-HT, 5-hydroxytryptamine; DA, dopamine; NE, norepinephrine; GABA, gamma-aminobutyric acid; BDNF, brain derived neurotrophic factor; NGF, nerve growth factor; MDA, malondialdehyde; SOD, superoxide dismutase; CRH, corticotropin-releasing hormone; TRH, thyrotropin-releasing hormone; GnRH, gonadotropin-releasing hormone; ACTH, adrenocorticotropic hormone; TSH, thyroid stimulating hormone; LH, luteinizing hormone; FSH, follicle-stimulating hormone; HPA, hypothalamus-pituitary-adrenal; HPT, hypothalamic-pituitary-thyroid; HPG, hypothalamic–pituitary–gonadal.

The causes of depression are complex, including genetic conditions, endocrine, mental state, living habits, and health status (15–17). Although the pathogenesis is complicated and remains elusive, several hypothesis/theories have been proposed to explain clinical manifestations from different perspectives. The pathology of depression was summarized in Figure 1 and elaborated as follows:

### 2.1 Monoamine hypothesis

Joseph J. Schildkraut proposed monoamine hypothesis as early as in 1965. The monoamine hypothesis describes that depression is resulted from the abnormal transmission of monoamine neurotransmitters, including synaptic deprivation of monoamine neurotransmitters, and dysfunctions of monoamine transporter and receptors (18, 19). Monoamine theory guided scientists to develop a number of antidepressant drugs including monoamine oxidase inhibitor isoniazid isopropylhydrazide although the drug was originally used to tuberculosis (6). Indeed, 80% of the antidepressant drugs that were approved by the United States Food and Drug Administration (FDA) target monoamine transmitter systems (20). The therapeutic effects of such drugs somehow approved monoamine hypothesis. The tricyclic drug tianeptine is known to promote serotonin reuptake and exhibit similar antidepressant effect as selective serotonin reuptake inhibitor (SSRI). However, some patients feel worse after taking tianeptine (21). Such clinical phenomena challenged monoamine hypothesis. The changes in monoamine levels appear to be the consequences other than the causes of depression.

### 2.2 Glutamatergic hypothesis and GABAergic deficit hypothesis

Glutamate is an excitatory amino acid that plays an essential role in cognitive functions such as learning and memory. Clinical studies observed a higher level of plasma glutamate in patients with depression (22). Indeed, N-methyl-D-aspartate receptor (NMDA-R) antagonists showed the potency of relieving depression symptoms (23). Thus, the glutamate hypothesis was proposed to highlight the elevation of glutamate between synapses as the causes of mental and emotional disorders. Accordingly, plasma glutamate level of patients is positively correlated with the severity of the disease (24). The inhibition of glutamate receptors became a therapeutic target for the development of novel antidepressant drugs. Interestingly, glutamate supplement exhibited antidepressant effects in some cases (25).

On the other hand, γ-aminobutyric acid (GABA) is synthesized from glutamate. Unlike glutamate, GABA is an inhibitory neurotransmitter. Under physiological conditions, the excitatory glutamate and the inhibitory GABA form a balance in the brains. GABA prevents the neurotoxicity of excess glutamate and termination of stress response (26). Depression patients and animal models suffered from the decreased levels of GABA and GABA-A receptor expression. Brexanolone alleviated postpartum depression by increasing GABA level and motivating the GABA-A receptor, suggesting the GABAergic deficit hypothesis (27, 28). Thus, depression may be caused by different pathological changes while the excitatory-inhibitory imbalance should be the common cause.

### 2.3 Hormone dysregulation

Hypothalamic-pituitary-adrenal (HPA) axis mainly regulates stress response. Under negative emotions or stress, HPA axis remains active. The hypersecretion of cortisol (corticosterone in rodents) causes neuronal damage and structural disturbances in the hippocampus, resulting in depression symptoms (29). Down-regulation of receptor induces the weakening of negative feedback while aggravates HPA axis excitement, forming a vicious circle. Similarly, the depression process involves other hormone systems, such as hypothalamic-pituitary-gonadal (HPG) axis and hypothalamic–pituitary–thyroid (HPT) axis.

Two third of depression patients are female, largely due to the frequent fluctuation of sex hormones in addition to environmental and genetic factors (3, 30). Both aging men and women are prone to mood disorders with the change of corresponding sex hormone levels, but exhibit different clinical outcomes (31). Females respond to stress in more sensitive manner than males as the sex hormones decline (32). Possibly due to the more influential role of estrogen in mood regulation, women usually become emotionally fragile during the low-estrogen period (33). Estrogen not only modulates cognition and emotion in the brain, but also exhibits neuroprotective effect (33, 34). Surprisingly, males with higher estrogen level tend to suffer from depression (35). Thus, caution is needed to address hormone dysregulation in depression in both sexes.

Stress is known to increase cortisol level and subsequently decrease the release of thyroid stimulating hormone (TSH) (36). Patients with bipolar II depression and anxiety disorder exhibit a lower TSH level and less response to thyrotropin-releasing hormone (TRH), while emotion also influences thyroid hormones (37, 38). People with thyroid disease are commonly associated with mood disorders (39, 40). Hyperthyroidism induces anxiety and irritability, whereas hypothyroidism causes depression. Consequently, thyroid supplementation may be used in the clinical treatment of depression (41).

### 2.4 Neurogenesis and neuroplasticity hypothesis

Depression is an emotional disease and may show signs at the cell and organ levels. Neuroanatomy studies revealed that hippocampus volume appeared to be reduced in the brains of depression patients (42). Bipolar patients was found to have less gray matter volume (43). Such changes may be caused by the decline of neurotrophic factors, such as brain-derived neurotropic factor (BDNF), nerve growth factor (NGF), and glia-derived neurotropic factor (GDNF) (44).

### 2.5 Miscellaneous theories

Scientists proposed several other conjectures of depression including inflammation theory, gut microbiota theory, glial pathology theory, epigenetic theory, infection theory, and “dys-stress” theory (45, 46). These theories together provided a comprehensive perspectivity to explain the depression mechanisms.

### 2.6 Current antidepressants and limitations

FDA-approved antidepressant drugs for adults are divided into seven categories: selective serotonin reuptake inhibitors (SSRIs), serotonin and norepinephrine reuptake inhibitors (SNRIs), tricyclic and tetracyclic antidepressants (TCAs and TeCAs), atypical antidepressants, monoamine oxidase inhibitors (MAOIs), N-methyl D-aspartate (NMDA) antagonist, neuroactive steroid, gamma-aminobutyric acid (GABA)-A receptor positive modulator (20). TCAs and MAOIs belong to the first generation of antidepressants with relatively strong short-term efficacy and low price (47). However, due to the severe side effects, these drugs are not considered the first choice for treating depression. SSRIs and SNRIs are considered as the first-line medications in clinical practice although side effects exist (9).

Most of synthetic antidepressant drugs are known to frequently cause severe side effects and exhibit symptoms including dizziness, nausea, weight change, sexual dysfunction, and apathy (48). The classification and common side effects of antidepressants are shown in the Table 1.

**TABLE 1:** Molecular targets and side effects of synthetic antidepressant drugs.

Class	Brand name	Generic name	Known targets	Side effects
SSRIs	Celexa	Citalopram	SLC6A4 inhibitor	Nausea, tremor, nervousness, problems sleeping, sexual problems, sweating, agitation, feeling tired
	Lexapro	Escitalopram	SLC6A4 inhibitor	
	Luvox	Fluvoxamine*	SLC6A4 inhibitor	
	Paxil Paxil CR Pexeva	Paroxetine	SLC6A4 inhibitor	
	Prozac	Fluoxetine	SLC6A4 inhibitor	
	Trintellix	Vortioxetine	SLC6A4 inhibitor; HTR1A agonist; HTR3A, HTR7 antagonist; HTR1B partial agonist	
	Viibryd	Vilazodone	SLC6A4 inhibitor; HTR1A agonist	
	Zoloft	Sertraline	SLC6A4 inhibitor	
SNRIs	Cymbalta	Duloxetine	SLC6A4, SLC6A2 inhibitor	Nausea, vomiting, dry mouth, constipation, fatigue, feeling drowsy, dizziness, sweating, sexual problems
	Effexor Effexor XR	Venlafaxine	SLC6A4, SLC6A2 inhibitor	
	Fetzima	Levomilnacipran	SLC6A4, SLC6A2 inhibitor	
	Pristiq Khedezla	Desvenlafaxine	SLC6A4, SLC6A2 inhibitor	
TCAs and TeCAs	Asendin	Amoxapine	SLC6A4, SLC6A2 inhibitor	Dry mouth, constipation, blurred vision, drowsiness, low blood pressure
	Elavil	Amitriptyline	SLC6A4, SLC6A2 inhibitor; HTR2A antagonist	
	Ludiomil	Maprotiline*	SLC6A2 inhibitor	
	Norpramin	Desipramine	SLC6A4, SLC6A2 inhibitor; HTR2A antagonist	
	Pamelor	Nortriptyline	SLC6A4, SLC6A2 inhibitor; HTR2A antagonist	
	Sinequan	Doxepin	HRH1, HRH2 antagonist; SLC6A4, SLC6A2 inhibitor	
	Surmontil	Trimipramine	SLC6A4, SLC6A2 inhibitor	
	Tofranil	Imipramine	SLC6A4, SLC6A2 inhibitor	
	Vivactil	Protriptyline	SLC6A4, SLC6A2 inhibitor	
Atypical antidepressants	Desyrel	Trazodone	SLC6A4 inhibitor; HTR1A antagonist and partial agonist; HTR2A antagonist; HTR2C agonist	Dry mouth, dizziness, blurred vision, feeling drowsy or sleepy, constipation feeling drowsy or sleepy, weight gain, dizziness, constipation, nausea, vomiting, blurred vision
	Serzone	Nefazodone	SLC6A4, SLC6A2 inhibitor; HTR1A, HTR2A, HTR2C antagonist; ADRA1	
	Remeron	Mirtazapine	HTR2A, 5HT3, ADRA2A antagonist	
	Wellbutrin Wellbutrin SR Wellbutrin XL	Bupropion	SLC6A3, SLC6A2 inhibitor	
MAOIs	Amira Aurorix	Moclobemide*	MAOA antagonist and inhibitor	Nausea, dry mouth, constipation, diarrhea, insomnia, dizziness, anxiety, restlessness nausea, restlessness, problems sleeping, dizziness, drowsiness
	Emsam (skin patch)	Selegiline	MAOB inhibitor	
	Marplan	Isocarboxazid	MAOA, MAOB inhibitor	
	Nardil	Phenelzine	MAOA, MAOB antagonist	
	Parnate	Tranylcypromine	MAOA, MAOB inhibitor	
NMDA antagonist	Spravato (nasal spray)	Esketamine	NMDAR	Dissociation, dizziness, nausea, sleepiness, spinning sensation, decreased feeling or sensitivity, anxiety
GABA-A receptor positive modulator	Zulresso (intravenous infusion)	Brexanolone	GABR	Sedation (tiredness), dry mouth, loss of consciousness, flushing

Information comes from FDA, Drugbank, KEGG. *Fluvoxamine: Also used to treat COVID-19; maprotiline: TeCAs, others in this class are TCAs; moclobemide: Didn’t been approved by FDA yet.

Indeed, the existing antidepressants are limited by different other factors including efficacy, patient compliance, withdrawal reaction and recurrence. As for efficacy, antidepressants often need at least 2 weeks to take effect (49). Many patients may feel the improvement of symptoms after taking medication but are not satisfactory with the overall effect while some patients may be getting even worse (6, 50). As for patient compliance, compliance with antidepressants is extremely poor. Quite a portion of patients are unwilling to follow antidepressant treatment (51). The fear of side effects is a key reason for poor compliance. As for the withdrawal reaction, more than half of the patients experience withdrawal symptoms, including gastrointestinal symptoms, flu-like symptoms, sleep disorders, sensory disorders, movement disorders, and emotional disorders (8). Some patients may have severe symptoms. Finally, the recurrence is also an important problem. Patients may be considered as fully cured by antidepressant treatment but more likely have depression again than normal people (52). Indeed, a quarter of patients relapse depression (53). Therefore, there is a strong need for other complementary or alternative therapies. It is believed that herbal remedies possess better potential than different physiotherapies and psychotherapies.

## 3 Herbal medicines for the treatment of depression

**FIGURE 2:**
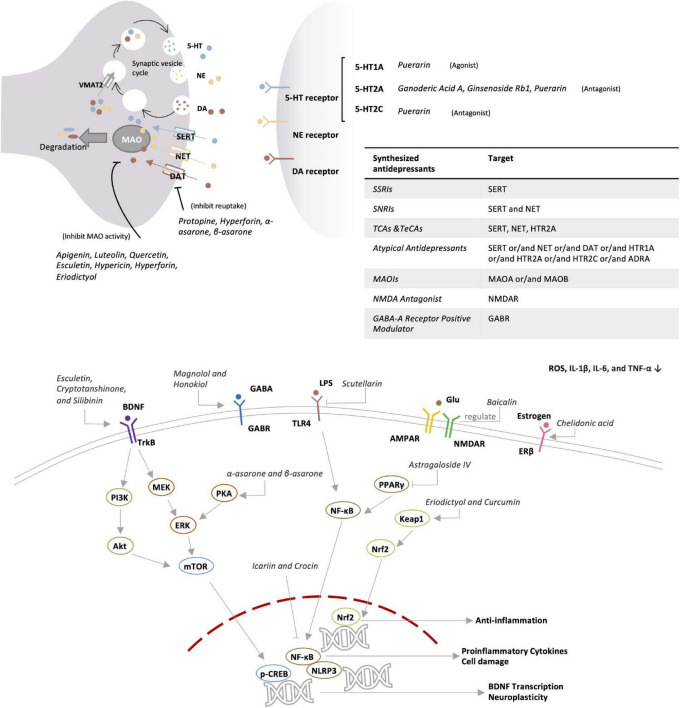
Potential antidepressant mechanism of botanical drugs.

Herbal ingredients are often used in combination. Presumably, different ingredients may act on several mechanisms in a coordinated manner. For example, hypericin, hyperforin, and eriodictyol may contribute to the antidepressant effects of *Hypericum perforatum* L. by targeting different mechanisms (54–57). On the other hand, some ingredients may act on more than one target. For example, puerarin not only acts on the 5-HT system and neurotransmitters but also regulates antioxidant and anti-inflammatory pathways, remodels gut microbiota, and modulates the HPA-axis (58–64). In this review, major ingredients and the related antidepressant mechanisms were searched from the recent literatures *via* PubMed and Google Scholar and summarized in Table 2 and Figure 2. In fact, different active compounds might act on one or several target proteins involved in the regulation of neurotransmitter function, HPA axis, BDNF signaling pathway, anti-inflammatory response, oxidative stress, intestinal microbiota and ferroptosis.

**TABLE 2:** Active constituents and molecular targets of herbal medicines.

Herbal source	Active constituents	Model	Depression model	Administration	Mechanism of action	References
*Acori tatarinowii* Rhizoma	α-asarone; β-asarone	Primary astrocytes from rat	N/A	15, 30, 50 μM	Increase synthesis and release of neurotrophic factors (NGF, BDNF, and GDNF)	(121)
	α-asarone	Adult male, Institute of Cancer Research (ICR) mice of age 8–10 weeks	AMPT (100 mg/kg, i.p., a catecholamine synthesis inhibitor)	20 mg/kg, i.p. (4 h after AMPT administration)	modulate α1 and α2 adrenoceptors, 5-HT1A receptors	(122)
*Albizia julibrissin* flower	SAG; SBG lignan glycosides	HeLa cells	N/A	10 μM SAG or 16 μM SBG	Non-competitively inhibit serotonin transporter	(123)
	SAG	8-week-old male Sprague-Dawley (SD) rats	Acute restraint-stressed	3.6 mg/kg, 7 days, p.o.	Modulate HPA axis and monoaminergic systems	(124)
*Alpinia officinarum* Hance	Hydroalcoholic extract	Male BALB/c mice	Daily chronic unpredictable stress (CUS), 3 weeks	50 and 100 mg/kg/day, 21 days, i.p.	Antioxidation	(125)
	Galangin	*In vitro* enzyme inhibition and binding test			Inhibit MAO-A and MAO-B	(126)
*Angelica sinensis* (Oliv.) Diels	75% ethanol extract	Male SD rats (weighing 180 ± 20 g)	Chronic unpredictable mild stress (CUMS), 3 weeks	3.6 and 7.2 g/kg.	Modulating the hematological anomalies	(127)
	75% ethanol extract	Male SD rats weighing 140–160 g	Chronic unpredictable mild stress (CUMS), 5 weeks	1 g/kg	Activating the BDNF signaling pathway (BDNF-ERK 1/2-CREB) and upregulating the hippocampal BDNF, p-ERK 1/2 and CREB expression.	(128)
	Z-ligustilide	Male SD rats (weight, 160–200 g; age, 7 weeks)	CUMS 35 days	20 and 40 mg/kg, 12 days, i.p.	Upregulate progesterone and allopregnanolone	(129)
*Apocynum venetum* L.	*Apocynum venetum* leaf extract	Adult male Wistar rats (42 days old) weighing 180–220 g	CUMS, 8 weeks	30, 60, and 125 mg/kg, 4 weeks, i.g.	Antioxidation, reduced hippocampal neuronal apoptosis, and enhanced hippocampal BDNF levels	(130)
Astragalus	Astragaloside IV	Male ICR mice, weighing 23–26 g	Repeated restraint stress (RRS)-induced mice, 9 days	16, 32, and 64 mg/kg/d, 12 days, i.g.	Anti-inflammation (*via* PPARγ/NF-κB/NLRP3 inflammasome axis)	(131)
			Lipopolysaccharide (LPS)-induced mice, 1 mg⋅kg-1⋅d-1, i.p., 2 days	20, 40 mg/kg/d, 14 days, i.p.		
*Atractylodes macrocephala* Koidz.	Atractylenolide III	Male SD rats (weighing 260–280 g on arrival)	CUMS, 28 days	3, 10, and 30 mg/kg, 14 days, p.o.	Anti-inflammation	(132)
*Camellia sinensis*	L-Theanine	Patients with MDD (four males; mean age: 41.0 ± 14.1 years, 16 females; 42.9 ± 12.0 years)		250 mg/day, 8 weeks	Blocking glutamate receptor	(80)
*Capsicum annuum* L. (Chili pepper)	Capsaicin	Four-week-old male C57BL/6J mice (bodyweight: 16–18 g)	0.052/0.104/0.208/0.415/ 0.83 mg/kg LPS, 5 days, i.p.	0.005% capsaicin in standard laboratory chow plus, 4 months	Regulation of 5-HT and TNF-α; remodeling gut microbiota	(133)
*Centella asiatica* (L.) Urban	Triterpenes	Male albino Wistar rats, aged 8–10 weeks and weighing 180–220 g	CUMS 8 weeks	Extraction 400 and 800 mg/kg, 8 weeks, p.o.	Upregulation of 5-HT, NE, and DA; regulation of HPA-axis	(134)
*Acori tatarinowii* Rhizoma	α-asarone; β-asarone	Primary astrocytes from rat	N/A	15, 30, 50 μM	Increase synthesis and release of neurotrophic factors (NGF, BDNF, and GDNF)	(121)
*Chelidonii herba*	Chelidonic acid	Male ICR mice (3 weeks old, 10–12 g)	N/A	0.02, 0.2, and 2 mg/kg, 14 days, p.o.	Upregulation of hippocampal 5-HT, dopamine, NE, and BDNF; anti-inflammation	(135)
*Citrus unshiu*	Peel extract	Male ICR mice (9-week-old, weighing 20–25 g)	Dexamethasone 40 mg/kg, 7 days, i.p.	30, 100, and 300 mg/kg, 14 days, p.o.	Modulate BDNF/TrkB/CREB signaling	(135)
		SH-SY5Y cells	dexamethasone 200 μM	10, 50, or 100 μg/mL		
*Cornus officinalis* (Cornus)	Loganin	Adult male Wistar rats, weighing 200–250 g	Depression and anxiety-like diabetic rats	40 mg/kg, 10 days, p.o.	Anti-inflammation	(136)
	Cornusfural B	PC12 cells	500 μM corticosterone, 24 h	10 μM, 24 h	Neuroprotective effects	(137)
	Morroniside	SD rats (220 ± 10 g, 7 weeks old)	immobilization stress, 14 days	Extract 100 mg/kg, 14 days, i.g.	Antioxidation (Blocked the MAPK/COX-2 Signaling Pathways in Rat Hippocampus)	(138)
		SH-SY5Y cells	300 μM H_2_O_2_, 24 h	Extract 20, 50, and 100 μg/mL, pretreat 2 h	Alleviated H_2_O_2_-Induced Apoptosis; enhance SOD, CAT, BDNF expression	
*Crocus sativus* L. (Saffron)	Crocin	Male Balb/cJ mice (18–24 g, 8–10 weeks of age)	CUMS, 7 weeks	30 mg/kg, 4 weeks, i.g.	Modulate HPA-axis,	(139)
		PC12 cells	CORT (200 μM), 24 h	12.5, 25, and 50 μM, pretreat 1 h	Upregulation of pituitary adenylate cyclase-activating polypeptide (PACAP) expression and phosphorylation of CREB and ERK	
		Six-week-old male C57BL/6 J mice	Chronic restraint stress (CRS)-induced	40 mg/kg, 6 weeks, p.o.	Modulate gut microbiota composition; reduced low-grade inflammation in the colon; reverse the decrease of fecal short-chain fatty acids (SCFAs)	(140)
		Six-week-old male C57BL/6 J mice	Corticosterone 20 mg/kg, 4 weeks, s.c.	20 and 40 mg/kg, 2 weeks, i.g.	Antioxidation (stimulate SIRT3 pathway); anti-inflammation	(141)
*Curcuma longa* L.	Curcumin	Male SD rats (180–220 g)	CUMS 28 days	100 mg/kg/d, 28 days, i.g.	Antioxidation (*via* Nrf2-ARE signaling pathway)	(103)
		SD rats (male, weight: 180–220 g, age: 40–45 day)	CUMS 6 weeks	100 mg/kg/d, 6 weeks, i.g.	Modulate PGC-1α/FNDC5/BDNF signaling pathway	(142)
*Cyperus rotundus* L.	α-cyperone	Male adult C57BL/6 mice	CUMS 5 weeks	5 and 10 mg/kg, 5 weeks, i.g.	Enhance neuroplasticity (*via* SIRT3/ROS/NF-κB pathway); suppressing NLRP3 inflammasome	(143)
Epimedii Herba	Icariin; icaritin	Male, 7-week-old C57 BL/6J mice	Social defeat (SD) stress 10 days	20 mg/kg, 4 weeks, p.o.	Anti-inflammation; regulation of BDNF: suppressing HMGB1-RAGE signaling, activating TLR4-NF-κB signaling	(144)
*Fraxinus rhynchophylla*	Esculin; esculetin; fraxin	Seven-week-old male c57BL/6 mice	Reserpine 0.5 mg/kg, 10 days, i.p.	50 mg/kg, 10 days, p.o.	Anti-inflammation; upregulate pCREB/BDNF expression	(145)
*Fructus arctii*	Arctigenin	Adult male C57BL/6 (WT B6) mice (8–10 weeks old, 18–22 g)	CUMS 6 weeks	25, 50, or 100 mg⋅kg	Anti-inflammation (*via* HMGB1/TLR4/NF-κB and TNF-α/TNFR1/NF-κB signaling pathways); decrease neuronal apoptosis; increase serum levels of 5-HT and dopamine	(146)
	Arctiin	Male C57BL/6 mice (18–22 g weight, 9 weeks old)	CUMS 8 weeks	25, 50 mg/kg, 4 weeks, i.g.		(147)
*Morus macroura* Miq. (Mulberry)	Ethanol extracts	Male rats	Post-myocardial infarction (MI) depression	200 mg/kg, 21 days, p.o.	Antioxidation; increase serotonin, dopamine, GABA, and ATP brain levels	(148)
Ganoderma	Polysaccharides	Male C57BL/6 mice 7–8 weeks old	Chronic social defeat stress (CSDS), 10 days	1 mg/kg, 5 mg/kg, and 12.5 mg/kg, 6 days	Mediate Dectin-1 receptors; enhanced AMPA receptor synaptic plasticity; anti-inflammation	(149)
	Ganoderic acid A	The SD rats (male; 240–260 g)	post-Stroke depression (CUMS 3 weeks)	10, 20, and 30 mg/mL, i.v.	Anti-inflammation (*via* the ERK/CREB pathway)	(150)
*Hedyotis corymbosa*	Ethanol extracts	SD rats (male; body weight—250–275 g)	Olfactory bulbectomy induced depression	50, 100, and 200 mg/kg, 14 days, p.o.	Upregulation of BDNF; regulation of HPA-axis; upregulation of 5-HT	(151)
*Hericium erinaceus*	Erinacine A	Male ICR mice weighing 20–25 g	Restraint stress 4 weeks	Extract 100, 200, and 400 mg/kg, 4 weeks, p.o.	Increase BDNF expression (*via* PI3K/Akt/GSK-3β pathway)	(152)
	Ethanol extract	PC-12 cells	400 μM corticosterone 24 h	0.125, 0.25, 0.5, and 1 mg/ml	Antioxidation	(153)
*Hypericum perforatum* L.	Hypericin	Female SD rats, 180–220 g	Postpartum depression	6.12 mg/kg, 42 days, i.g.	Anti-inflammation; up-regulate the estrogen receptor (ER) expression; reduce the level of CORT (*via* reversing the activity of 11β-HSD2 enzyme)	(154)
	Hyperforin	Male C57BL/6 J mice (7 weeks old)	CUMS 8 weeks	2.5 and 5 mg/kg, 45 days, i.p.	Regulate BDNF pathway and zinc homeostasis	(56)
	Eriodictyol	Male SD rats weighting 240–260 g	LPS 1 mg/kg, 2 days, i.p.	10, 30, and 100 mg/kg, 28 days, i.g.	Anti-inflammation; anti-oxidation (*via* Nrf2/HO-1 axis)	(102)
			CUMS 28 days			
*Lavandula angustifolia* Mill (Lavender)	Essential oil	Male SD rats weighing 240–260 g (6–7 weeks of age)	40 mg/kg corticosterone, 14 days, s.c.	Exposed to a cotton saturated with 2.5% LEO, 14 days	Upregulation of BDNF and oxytocin	(155)
*Leonurus japonicus* Houtt	Leonurine	Male C57BL/6 (8–10 weeks) mice with a body weight of 18–22 g	Chronic mild stress (CMS) 10 weeks	30 and 60 mg/kg, 4 weeks, i.g.	Improvement of monoamine neurotransmitters (5-HT, NE, and DA); anti-inflammation	(156)
		PC12 cells	300 μM CORT 24 h	10, 20, 40, 60, 80, and 100 μM, pretreat 2 h	Neuroprotective effects (*via* GR/SGK1 signaling pathway)	(157)
*Magnolia officinalis*	Honokiol	Male SD rats, weighing 200–220 g	CUMS 28 days	10 mg/kg Honokiol, 21 days, i.g.	Regulate HIF-1α-VEGF signaling pathway, VEGFR-2-mediated PI3K/AKT/mTOR signaling pathway	(158)
		PC 12	N/A	2, 5, 8, 10, and 16 μM, 24 h/48 h	Regulate HIF-1α-VEGF signaling pathway	
	Magnolol	Female C57BL/6J mice (18–22 g)	CUMS 7 weeks	50 and 100 mg/kg, 3 weeks, i.g.	Inhibit M1 microglia polarization and promoted M2 microglia polarization *via* Nrf2/HO-1/NLRP3 signaling	(159)
		BV2 cells	LPS (1 μg/ml) + ATP (20 μM) 24 h	(5, 10, and 20 μM) 2 h prior		
*Morinda officinalis*	Fructooligosaccharides	Male SD rats (160 ± 20 g, 6-week-old)	CUMS 7 weeks	50 mg/kg, 3 weeks, i.g.	Remodel gut microbiota; decrease urine and plasma corticosterone	(160)
*Monodora myristica* (Gaertn.)	Essential oils	Male Wistar rats (150–180 g)	CUMS 5 weeks	150 and 300 mg/kg, 5 weeks, p.o.	Decrease serum CORT and brain MAO-A levels	(161)
Nelumbinis semen	Neferine	C57BL/6J mice (6-week-old, male)	CUMS 8 weeks	20 mg/kg, 4 weeks, i.p.	Remodeling gut microbiota	(162)
*Paeonia lactiflora* Pall.	Albiflorin	Male SD rats (180–200 g)	CUMS 8 weeks	7 and 14 mg/kg, 14 days, p.o.	Remodel gut microbiota; inhibit D-amino acid oxidase	(163)
		Male ICR mice (18–22 g)	4 mg/kg reserpine, i.p.	7 and 14 mg/kg, 7 days, p.o.		
	Paeoniflorin	Male C57BL/6 J mice weighted at 19–23 g at 6–9 weeks	CRS 5 weeks	10, 30, and 60 mg/kg, 5 weeks, i.p.	Affect the ERK1/2 pathway	(164)
*Panax ginseng* C. A. Mey.	Ginsenoside Rg1	Male SD rats (weight: 200–250 g)	CRS 28 days	20 mg/kg, 28 days, i.g.	Regulate GAS5/EZH2/SOCS3/NRF2 Axis	(165)
	Ginsenoside Rb1	CD1 (12 months old, male) and C57BL/6J (7–8 weeks old, male) mice	CSDS 28 days	35 and 70 mg/kg, 28 days, i.g.	Regulate BDNF–TrkB signaling pathway	(166)
*Perilla frutescens*	Volatile oil	Female SD rats (180–200 g)	Menopausal depression (ovariectomy + CUMS 14 days)	10.8, 32.4, and 97.2 mg/kg, 14 days, i.g.	Regulate metabolites	(167)
Platycodins folium	Extract	Adult male ICR mice, weighing 20 ± 2 g	LPS (0.83 mg/kg), 24 h, i.p.	100, 200, and 400 mg/kg, 7 days, i.g.	Regulation of several metabolic pathways	(168)
*Rhizoma polygonati*	Polysaccharide	Male C57BL/6 mice (3 months old, 20–25 g)	LPS (2 mg/kg), 24 h, i.p.	100, 200, and 400 mg/kg, 10 days, i.g.	Anti-inflammation; reduce ROS/HPA axis hyperfunction	(169)
			CUMS 35 days	400 mg/kg, 35 days, i.g.		
Pueraria Lobelia	Puerarin	SD rats (male, 200 ± 20 g)	CUS 28 days	30, 60, and 120 mg/kg, 10 days	Regulate monoamine neurotransmitter; regulate HPA-axis; regulate HPG-axis	(60)
		male C57BL/6N mice (7–8 weeks, 18–25 g)	LPS (0.083 mg/kg) 24 h	30, 60, and 120 mg/kg, 25 h, i.g.	Anti-inflammation; inhibited the RagA/mTOR/p70S6K pathway	(63)
		Highly Differentiated PC12 Cell	LPS (200 ng/ml) 24 h	10, 25, and 50 μM, 24 h		
		Male SD rats (160–180 g)	High-fat diet (HFD)/CUMS 11 weeks	30, 60, and 120 mg/kg, 7 days	Inhibit TLR4-associated inflammatory responses	(64)
Radix Bupleuri	Saikosaponin A	Female Wistar rats (36-week old and 350–370 g weight)	CUMS 8 weeks	25, 50, or 100 mg/kg, 4 weeks, p.o.	Up-regulation of the BDNF-TrkB signaling pathway; anti-inflammation; regulation of HPA-axis	(170)
	Saikosaponin-d	Male ICR mice, 5 weeks old, weighing 20–22 g	LPS with increasing dose (0.052/0.104/0.208/0.415/ 0.83 mg/kg), 4 days, i.p.	0.5 and 1 mg/kg, 2 weeks, i.g.	Mitigate LPA1-mediated neuronal apoptosis; attenuate LPS-induced activation of RhoA/MAPK/NFκB signaling pathway	(171)
		SH-SY5Y	LPA (4 μM)/LPS (1 μg/ml)	0.5, 1, and 2 μM)		
*Rehmannia glutinosa*	Catalpol	Adult male Kunming mice (weighing 18–22 g, 3–4 weeks old)	Depressive-like behavior of STZ (streptozocin)-induced hyperglycemia models	5, 10, and 20 mg/kg, 21 days, i.g.	Antioxidation (*via* PI3K/AKT/Nrf2/HO-1 signaling pathway)	(172)
Rhubarb	Emodin	8-week-old male SD rats	CUMS 7 weeks	80 mg/kg, i.g.	Anti-inflammation (targeting miR-139-5p/5-LO)	(173)
*Salvia miltiorrhiza*	Cryptotanshinone	Male C57BL/6 mice (8 weeks, 20–25 g)	CUS 14 days	20 mg/kg, 14 days, i.g.	Anti-inflammation (*via* NF-κB signaling pathway); restore hippocampal neurogenesis (*via* BDNF/TrkB pathway)	(174)
Santalum album seeds	Extract	Ten weeks old Male Swiss mice weighing 20 to 25 g	Cecal ligation and puncture (CLP) model	100 and 200 mg/kg, 24 h	Antioxidation	(175)
*Schisandra chinensis* Fructus	Schisantherin A	Male ICR mice, weighing 20 ± 2 g	N/A	1.75, 3.5, and 7 mg/kg, 7 days, i.g.	Regulate GABA/Glu system	(176)
	Schisantherin B	Male KM mice, 10-week old (20–25 g) and 11-month old (50–60 g)	Acute stress (FST)	15 mg/kg, 10 days, i.p.	Promote PI3K/AKT/mTOR pathway	(177)
	Gomisin A	N9 microglial cells	LPS (1 μg/ml) 24 h	1, 3, 10, 30, and 100 μM, pretreat 2 h	Inhibit TAK1-IKKα/β-IκB-NF-κB and MAPKs inflammatory signaling pathways; anti-oxidation	(178)
	Gomisin N	Seven-week-old male ddY mice	LPS 500 μg/kg, 24 h, i.p.	100 mg/kg, 25 h, p.o.	Anti-inflammation	(179)
		BV2 cells	LPS 0.1 μg/mL, 6 h	1.6–50 μM, 7 h		
*Scutellaria baicalensis* Georgi	Decoction (contain baicalin, baicalein, wogonoside, and wogonin)	Male SD rats (190–220 g)	CUMS 6 weeks	500 and 1,000 mg/kg, 3 weeks, i.g.	Regulate CREB and BDNF (*via* activating cAMP/PKA pathway)	(180)
	Wogonin; baicalein	N/A	N/A	enzyme assays	Inhibit MAO	(181)
	Baicalin	Adult male ICR mice (7–8 weeks, weighing 20–25 g)	CUMS 21 days	50 and 100 mg/kg, 21 days	Regulating neurogenesis (*via* Wnt/β-catenin pathway)	(182)
	Scutellarin	Male C57BL/6 mice (6–8-week-old)	LPS 0.83 mg/kg, 7th day, i.p	50 mg/kg, 9 days, i.p.	Anti-inflammation (*via* TLR4/NF-κB pathway)	(183)
*Silybum marianum*	Silibinin	Male SD rats (8 weeks old with a body weight of 220–350 g)	Single prolonged stress (SPS)	25, 50, and 100 mg/kg, 14 days, i.p.	Increase 5-HT synthesis; modulate monoamine levels (DA and NE)	(184)
	Silymarin	Swiss albino mice weighing 30–35 g (70–80 days old)	CUMS 28 days	100 and 200 mg/kg, 21 days, p.o.	Modulate HPA axis; antioxidation; anti-inflammation; increasing BDNF expression; modulate monoamines	(184)
*Ziziphus jujuba* Mill. seeds	Ethanol extract	Male ICR mice (6 weeks old, 30 ± 1 g)	CUMS 31 days	100 and 300 mg/kg, 28 days, p.o.	Upregulate 5-HT and NE (inhibit MAO-B and AChE); upregulate BDNF	(185)

### 3.1 Regulation of neurotransmitter function

#### 3.1.1 Targeting monoamine neurotransmitters system

Monoamine neurotransmitters include serotonin (5-HT), noradrenaline (NE), and dopamine (DA). 5-HT is an indole neurotransmitter and induces a happy mood in the brain (65). As two types of catecholamines. NE is an excitatory neurotransmitter and alerts people by producing excitement and anger, whereas DA is called the happiness hormone (66). The deficiency of these neurotransmitters results in apathy and the lack of energy. Unlike chemically synthesized antidepressants, herbal medicines may exhibit a broad spectrum of effects on the activity of multiple neurotransmitters. As a key herbal medicine antidepressant, hyperforin derived from St. John’s wort simultaneously inhibits the reabsorption of 5-HT, NE, and DA with similar effectiveness (54). Protopine reduces the reuptake of 5-HT and NE *via* inhibiting the transporter (67). Apigenin, luteolin, and quercetin from *Cayratia japonica* inhibit the activities of MAO-A and MAO-B (68). Highly like the current antidepressants, herbal medicines target 5-HT receptors as the main antidepressant mechanism. Puerarin derived from *Radix puerariae* acts not only as the antagonist for 5-HT2C and 5-HT2A receptors but also as the agonist for 5-HT1A receptor (58, 59).

#### 3.1.2 Targeting GABAergic system

GABA receptors have long been therapeutic targets for anxiety disorders. The current antidepressants improve depression in mice *via* regulating the GABA system and enhancing the activity of GABAnergic neurons (69). Anxiety and depression often co-exist and influence each other in clinical practice (70). GABA-A receptor positive modulator Zulresso was approved by FDA in 2019 as a treatment for postpartum depression (71). The bark of Magnolia officinalis is well-documented for treating depression in traditional Chinese medicine formulations, while honokiol and magnolol are considered as the active ingredients. Indeed, magnolol treatment reversed the depressive symptoms in rats after chronic unpredictable mild stress (CUMS). Following the treatment, CUMS rats performed equally well in the tests for sucrose preference, locomotor activity, and forced swimming test compared with the rats in the control group, indicating that magnolol may be equally effective as Fluoxetine hydrochloride (72). Honokiol and magnolol positively regulate GABA-A receptors, especially δ-containing receptors (73). It was recently found that GABA-B receptor inhibitors might be potential antidepressant drugs (74). Interestingly, GABA-B1 receptor knockout mice appeared to more anxious than wild breeds. Presumably, GABA-B receptor positive allosteric agents are anxiolytic whereas the antagonists could be antidepressants (75). Nevertheless, both inhibitors and agonists were found to exhibit an antidepressant effect (76).

#### 3.1.3 Targeting L-glutamate signaling pathway

Glutamate receptors include ionotropic and metabotropic forms for rapidly transmitting excitation and widely affecting neural function by coupling with G protein, receptively (77). Depressive symptoms could be relieved by N-methyl-d-aspartate (NMDA) receptor antagonists, group I metabotropic glutamate receptor (mGluR1 and mGluR5) antagonists, and positive modulators of α-amino-3-hydroxy-5-methyl-4-isoxazole propionic acid (AMPA) receptors (78). L-Theanine from *Camellia sinensis* share similar structure with glutamate and binds to several glutamate receptors, thereby blocking the action of glutamate and reducing glutamate excitotoxicity (79). After treatment for 8 weeks, L-theanine improved depressive symptoms including anxiety, sleep disturbance, and cognitive impairment in MDD patients (80).

### 3.2 Regulation of HPA-axis

The HPA-axis involves three hormones [i.e., corticotropin-releasing hormone (CRH), adrenocorticotropic hormone (ACTH), and cortisol] and mainly mediates stress in the human body (81). As a stress hormone, cortisol affects the levels of neurotransmitters such as 5-HT. Anti-glucocorticoid therapy benefits the brain’s reward mechanisms and alleviates depression (82). Many herbs, such as *Scutellaria baicalensis*, *Phellodendron phellodendri*, and Chuanxiong, are known to induce significant reduction of plasma corticosterone levels in depressed mice (83). Based on radiometric ligand-binding assays, icariin could restore the down-regulation of glucocorticoid receptor in social defeat mouse model of depression (84). Several flavonoids (e.g., hypericin, hyperoside, isoquercitrin, and miquelianin) from St. John’s wort significantly reduced the levels of ACTH and corticosterone in rats, and could achieve better effects than imipramine positive control (85).

### 3.3 Regulation of BDNF signaling pathway

Brain-derived neurotropic factor is known to regulate the growth and function of neuron cells and thereby plays an important role in the regulation of learning and memory (86). Stress reduces the level of BDNF in the brain, leading to atrophy and cell loss in hippocampus and prefrontal cortex, suggesting the link of BDNF with depression (87). Indeed, most of antidepressant drugs could booster the expression of BDNF (88). Peony glycosides from Radix Paeoniae Alba increased the BDNF mRNA level in the brain and improved depressive-behaviors in CUMS-induced mouse model of depression (89). Traditional Chinese medicine formulation PAPZ of four ingredients (i.e., Radix Ginseng, Radix Angelicae Sinensis, Radix Polygalae, and Semen Ziziphi Spinosae) increased the protein expression of BDNF and alleviated the depressive behavior in corticosterone-challenged mice (90). Esculetin from *Cichorium intybus* L. activated BDNF/TrkB pathway in LPS-depressed mice by increasing BDNF expression (91). BDNF was also found to enhance the function of 5-HT transporter and reduce the level of 5-HT in the synaptic cleft, indicating a need to investigate the cross-talks between different systems (92).

### 3.4 Regulation of anti-inflammatory response

Depression patients generally show marked increase in pro-inflammatory cytokines (e.g., CRP, IL-3, IL-6, and IL-12) (93). Indeed, anti-inflammatory drugs like celecoxib could effectively relieve the symptoms of depression (94). Many herbal medicines are well-documented for anti-inflammatory properties and potential in the treatment of depression in the inflammatory model of depression (95). *Crocus sativus* L. (Saffron) is an important medicinal ingredient and also a common spice in North African, Mediterranean, and Middle Eastern countries. As one of the main components, crocin improved depressive symptoms and reduced the expression of inflammatory cytokines (e.g., IL-1β, IL-18, and TNF-α) in the hippocampus of LPS-depressed mice (96). The cellular experiments found that crocin skewed the polarization of glial BV-2 cells from the inflammatory M1 phenotype to the M2 phenotype by inhibiting the NF-kB and NLRP3 signaling pathway (96). Esculetin as a coumarin compound in various plants exhibited strong anti-inflammatory effect, reduced the levels of IL-1β, IL-6, and TNF-α in serum and hippocampus, and down-regulated the hippocampal expression of iNOS and COX-2 in LPS-depressed mice (91). Moreover, BDNF exhibits anti-inflammatory effect, suggesting that the increase in BDNF level also represents an anti-inflammatory mechanism (97).

### 3.5 Regulation of oxidative stress

Oxidative stress is implicated in various neurodegenerative diseases including AD and PD (98). Depression patients often suffer from cognitive impairment, likely as the result of oxidative stress (99). The antioxidant system is likely disturbed in people with depression (100). Interestingly, 5-HT deficiency appeared to be associated with altered expression of antioxidant enzymes (101). Many herbal medicines are well-known for antioxidative effects and may relieve depression symptoms through antioxidant activity. Eriodictyol is a bitter-masking flavanone, a flavonoid derived from *Eriodictyon californicum*. Eriodictyol reduced oxidative damage, prevented cell apoptosis, induced glutathione synthesis, and reduced ROS production in H_2_O_2_-treated PC12 cells (57). On the other hand, eriodictyol profoundly ameliorated sucrose preference, reduced immobility time in forced swimming test and feeding latency in novelty-suppressed feeding test in LPS- and CUMS-induced rat model of depression (102). Turmeric is one of the raw materials of curry as a spice, and curcumin in it can restore the effects of oxidative stress and prevent depression caused by CUMS (103, 104). Polyphenols are found in many fruits and vegetables, and it has been suggested that diet therapy may be used to relieve depression (105).

### 3.6 Modulation of intestinal microbiota

The enteric nervous system (ENS) is known to control gastrointestinal behavior *via* the actions of neurons and neurotransmitters in a manner independent of central nervous system (CNS) input, thereby also known as the “second brain” (106). Indeed, intestinal flora directly produces neurotransmitters (e.g., serotonin and GABA), and regulates brain functions and emotion through the microbiota–gut–brain (MGB or BGM) axis (107, 108). Gut microbiota in the large intestine synthesize various short-chain fatty acids (SCFAs) as the major metabolites for modulating the levels of neurotransmitters and neurotrophic factors and directly affecting brain functions (109, 110). Probiotics Allobaculum and Bifidobacterium were considerably reduced in the gut of depressed patients (111). Interestingly, traditional Chinese medicine formulation Kaixinsan could increase the relative abundance of Allobaculum and Bifidobacterium in the gut of CUMS mice (112). The concurrent use of antibiotics decreased the antidepressant effect of Kaixinsan, suggesting the link of Allobaculum and Bifidobacterium with depression (112). Moreover, puerarin reversed stress-induced disruption of gut microflora *via* increasing the level of beneficial bacteria and decreasing the inflammatory bacteria in CUMS mouse (113). Collectively, herbal medicines might exhibit antidepressant activity by affecting gut microbiota.

### 3.7 Regulation of ferroptosis

Ferroptosis describes iron-mediated oxidative cell death, largely due to the toxicity from dramatical increase in the level of intracellular iron ions (114, 115). Ferroptosis has emerged as a hot target for cancer therapy in the past decade. Lipid peroxidation is hyperactive in the depressed population than in the normal population and tightly associated with ferroptosis, suggesting a new therapeutic target (116). A recent analysis of hippocampal proteomes identified the hyperactivation of ferroptosis pathway in CUMS mice (117). Interestingly, traditional Chinese medicine formulation Xiaoyaosan was shown to substantially reduce the total iron and ferrous content in the hippocampus from CUMS mouse model, possibly by regulating PEBP1-GPX4-mediated ferroptosis (118). Galangin, a polyphenolic compound from *Alpinia officinarum*, also inhibited ferroptosis in the hippocampus by activating the SLC7A11/GPX4 axis (119). Iron chelators and lipophilic antioxidants were suggested for preventing ferroptosis (120). Considering the number, chemical diversity and potency, herbal products represent a rich source for the discovery of new ferroptosis-targeting antidepressants.

### 3.8 Pathway enrichment analysis

**FIGURE 3:**
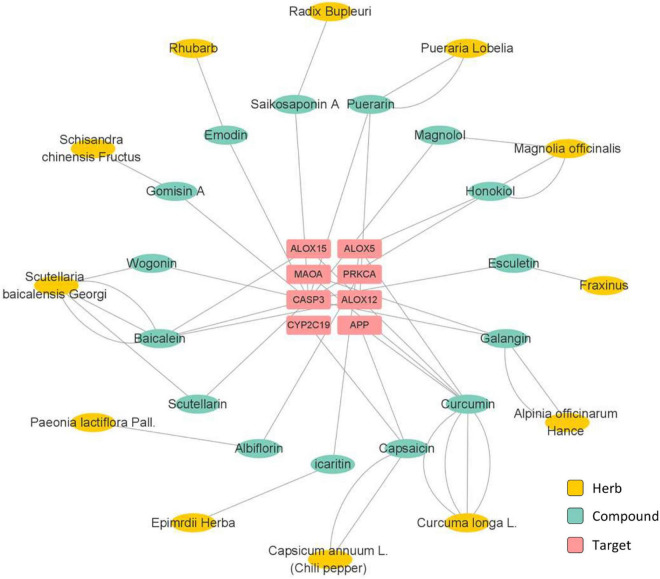
Network pharmacology analysis of herbs and the active compounds for targeting serotonergic synapse pathway.

**FIGURE 4:**
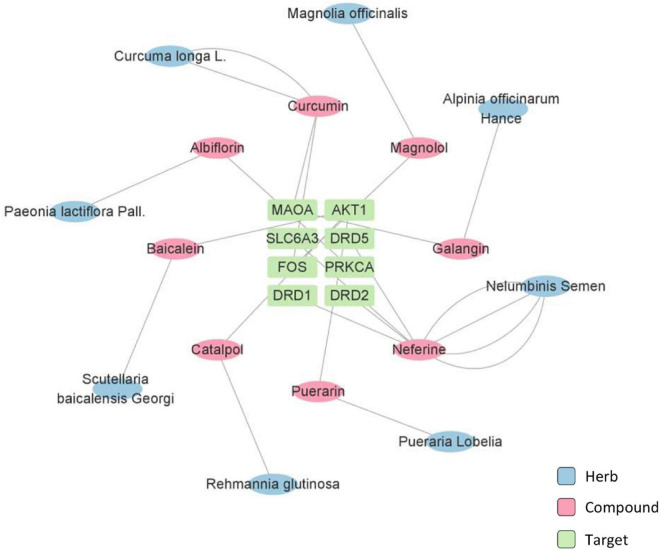
Network pharmacology analysis of herbs and the active compounds for targeting dopaminergic synapse pathway.

The potent active compounds were further examined through network pharmacology analysis while the target proteins were accordingly predicted (Figures 3, 4). Specifically, the prediction and screening of potential depression-related targets were performed using Similarity Ensemble Approach (SEA) at https://sea.bkslab.org, the Search Tool for Interactions of Chemicals (STITCH) at http://stitch.embl.de, SwissTargetPrediction at http://www.swisstargetprediction.ch, Therapeutic Target Database (TTD) at http://db.idrblab.net/ttd, Comparative Toxicogenomics Database (CTD) at http://ctdbase.org, PharmGKB at https://www.pharmgkb.org, DisGeNET at https://www.disgenet.org, and GeneCards at https://www.genecards.org. Kyoto Encyclopedia of Genes and Genomes (KEGG) Pathway Enrichment of selected targets were performed using the online bioinformatics tool DAVID at http://david.ncifcrf.gov. Interestingly, the pathway enrichment analysis suggests that active herbal compounds mainly target serotonergic synapse pathway (KEGG: map04726) and dopaminergic synapse pathway (KEGG: map04728) in relation to depression. As shown in Figure 3, eight targets (i.e., APP, CASP3, PRKCA, MAOA, ALOX12, ALOX15, ALOX5, and CYP2C19) were enriched for regulating serotonergic synapse pathway, whereas the most related compounds were curcumin from *Curcuma longa* L. and baicalein from *Scutellaria baicalensis* Georgi. As shown in Figure 4, eight targets (i.e., SLC6A3, AKT1, PRKCA, FOS, MAOA, DRD1, DRD2, and DRD5) were enriched for regulating the dopaminergic synapse pathway whereas neferine from Nelumbinis semen was mostly studied.

## 4 Conclusion

In this review, we initially discussed the current understanding on the pathology of depression and the molecular targets for different classes of synthetic drugs. Subsequently, we performed comprehensive review and network pharmacology analysis to understand the antidepressant activities of herbal medicines and reveal the underlying mechanisms. Herbal medicines appear to be effective for the treatment of depression without causing undesirable side-effects. As such, the present review may pave a new avenue for the development of novel antidepressant strategies.

## Author contributions

YS prepared the original draft. JR and JZ designed, reviewed, and revised the manuscript. JR supervised the work. All authors contributed to the article and approved the submitted version.
